# Effectiveness of *Persea major* Kopp (Lauraceae) extract against *Enterococcus faecalis*: a preliminary in vitro study

**DOI:** 10.1186/s13104-017-2443-x

**Published:** 2017-03-06

**Authors:** Lusiane Volpato, Marilisa Carneiro Leão Gabardo, Denise Piotto Leonardi, Paulo Henrique Tomazinho, Leila Teresinha Maranho, Flares Baratto-Filho

**Affiliations:** School of Health and Biological Sciences, Positivo University, Rua Prof. Pedro Viriato Parigot de Souza 5300, Curitiba, Paraná 81280-330 Brazil

**Keywords:** Medicinal plants, Antibacterial agents, Calcium hydroxide, Chlorhexidine

## Abstract

**Background:**

*Persea major* Kopp (Lauraceae) is a plant with wound healing, antibacterial, and analgesic properties. The aim of this study was to assess the in vitro antibacterial activity of the concentrated crude extract (CCE) and ethyl acetate fraction (EAF) of this plant against *Enterococcus faecalis* and compare it with calcium hydroxide [Ca(OH)_2_] paste and 2% chlorhexidine digluconate (CHX).

**Methods:**

The plant material was collected, and an extract was prepared according to the requirements of the study (CCE and EAF). The minimum inhibitory concentrations (MICs) of CCE, EAF, Ca(OH)_2_, Ca(OH)_2_ + CCE, and CHX against *E. faecalis* were determined using the broth microdilution method

**Results:**

The EAF inhibited *E. faecalis* at concentrations of 166.50, 83.25, and 41.62 mg mL^−1^, and 1.00, 0.50, and 0.25% of CHX solutions showed antimicrobial activity. The MICs of Ca(OH)_2_ paste were 166.50 and 83.25 mg mL^−1^, whereas Ca(OH)_2_ + CCE showed antimicrobial activity only at a concentration of 166.50 mg mL^−1^. CCE showed no inhibitory effect at any of the concentrations tested

**Conclusions:**

The CCE did not show any antimicrobial activity against *E. faecalis*; however, the EAF was the most effective among the three highest concentrations tested.

## Background

The aim of an endodontic treatment is to eliminate bacteria and their by-products from the root canal system [1]. The indiscriminate use of antibiotics has increased antimicrobial resistance, which results in the persistence infections and a decrease in the effectiveness of drug therapy [2]. In Endodontics, one of the pathogens associated with the development and maintenance of the infectious process and failure of therapy is *Enterococcus faecalis* [3].

The effect of substances against *E. faecalis*, as calcium hydroxide [Ca(OH)_2_] [4–6] and 2% chlorhexidine digluconate (CHX) [6] is limited, even when they are used in combination [7]. Alternative antimicrobial products and drugs of plant origin are promising candidates to overcome this problem. Previous studies have examined the effects of natural substances against *E. faecalis* [8–14]. *Persea major* Kopp (Lauraceae) is one with antimicrobial potential. It is native to Brazil, commonly known as “pau-andrade;” and has shown wound healing, antibacterial, and analgesic effects [15]. The species of the genus *Persea* are characterized by the presence of benzil tetrahydroisoquinolines alkaloids, flavonoids (keampferol and kaempferol-3-rhamnoside, quercetin and quercetin-3-rhamnoside; flavan-3,4-diol: leucocyanidins and flavan-3-ol: (±)-catechin) [16]. Other authors isolated and characterized the leaves of *Persea obovatifolia* and registered new neolignans including obovatifol [(2S,3S)-2,3-dihydro-2- (3,4-dihydroxy-5-methoxyphenyl)-7-methoxy-3-methyl-5-trans-propenyl benzofuran], obovaten [2-(3,4-dihydroxy-5-methoxyphenyl)-7-methoxy-3- methyl-5-trans-propenyl benzofuran], perseal C [(2S,3R)-2,3-dihydro-2-(3,4-methylenedioxyphenyl)-5- formyl-3-hydroxymethyl-7-methoxy benzofuran] and perseal D [2-(3,4-dihydroxy-5-methoxyphenyl)-5-formyl-7- methoxy-3-methyl benzofuran]. These new neolignans, P-388, KB16, A549, and HT-29, showed cytotoxic action against cancer cells [17]. The presence of tannins was found in *Persea americana* leaves [17–19] and in seeds [20, 21]. Phytochemical analysis showed that it has a number of compounds, especially the tannins, that precipitate proteins [22] and have antibacterial, antiseptic, antifungal, and hemostatic properties [23]. In Endodontics the use of derivatives from this plant is still unknown.

Therefore, this study aimed to examine the antimicrobial activity of extracts from the bark of *P. major* (concentrated crude extract, CCE and ethyl acetate fraction, EAF) against *E. faecalis* by using the broth microdilution technique followed by determination of the minimum inhibitory concentrations (MICs). In addition, we compared the antimicrobial effect of these extracts with that of Ca(OH)_2_, Ca(OH)_2_ + CCE, and 2% CHX.

## Methods

### Collection and identification of plant material

Samples of the bark of *P. major* were collected from an adult plant in the rural region of the municipality of Campo Largo, Paraná, Brazil. It was not necessary permission to carry out sampling. For the identification of the plant material, we created herbarium specimens and deposited them in the Herbarium of the Positivo University (Registry Number 100).

**Fig. 1:**
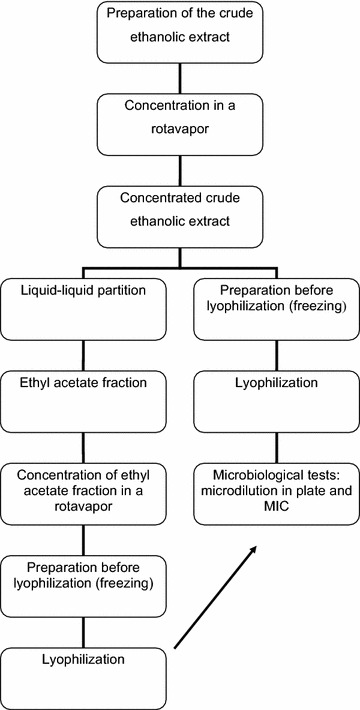
Steps used to assess the antimicrobial efficacy of the extracts from the *Persea major* Kopp (Lauraceae)

The steps for preparation and fractionation of extracts are presented in Fig. 1.

The plant material was dried in a forced air oven at 40 °C until the weight stabilized. Then, it was crushed to a powder, and thus, we obtained the pulverized drug for preparation of the crude ethanolic extract.

### Obtaining plant extracts

Plant extracts were obtained according to Younes et al. [24]. We blended the material by using an industrial turbine (Rodrimar, São Paulo, SP, Brazil). We used 187.11 g of the dried and pulverized bark and 2000 mL of 70% ethanol (Rioquímica, São José do Rio Preto, SP, Brazil). This material was extracted for 20 min and the liquid obtained was filtered in vacuum, resulting in the crude ethanolic extract (approximately 2050 mL).

The concentration of the extract was performed in a rotary evaporator (Quimis, Diadema, SP, Brazil). Each cycle lasted for approximately 30 min, and the rotation speed was adjusted to six to obtain the CCE.

### Fractionation of the CCE (liquid–liquid partition)

Liquid–liquid partition [25] was performed using the CCE and 2000 mL of ethyl acetate (Vetec, Duque de Caxias, RJ, Brazil). We placed 100 mL of the CCE in a separation funnel, and then, an equivalent volume of ethyl acetate (100 mL) was poured on it with gentle agitation. This step was repeated nine times, until the solvent was clear. Thus, we obtained the EAF, which was then concentrated in a rotary evaporator and stored in an amber flask.

### Freezing and lyophilization

The extracts were frozen in autoclaved plastic containers and covered with a plastic wrap for 48 h. Microperforations were introduced in the wrap using a sterilized gingival needle to allow the sublimation of the liquid. Then, the samples were placed in a lyophilizer (Ilsinh Lab. Co., Ltd., Korea). Any residual liquid was removed, and the sample was frozen again until the sample dried completely. The lyophilization process was completed in 7 days.

### Preparation of the drug for the analysis of antimicrobial activity

The samples were powdered using a glass rod. Visual inspection allowed the granulometric analysis of the preparations, which were weighed in mini Petri dishes in a precision balance. For preparing drug samples, 500 mg lyophilized powders from the CCE, EAF, and Ca(OH)_2_ P. A. (Biodinâmica, Ibiporã, PR, Brazil), and 0.1 mL of 2% CHX solution (chlorhexidine S 2%—FGM, Joinville, SC, Brazil), were used.

The propylene glycol was the vehicle for treatment of the powder samples and for experiments because of its harmless characteristics and the common use in endodontic treatments.

Two samples from *P. major* were very viscous, which hampered pipetting. Therefore, 0.05 mL of sterile saline solution were added to them, including the Ca(OH)_2_ paste. No vehicle was required for the 2% CHX solution.

### Determination of the MIC by using the broth microdilution technique

We used the *E. faecalis* strain (ATCC 19433) and the Mueller–Hinton broth as the culture medium, with a pH between 7.2 and 7.4, at 25 °C. The bacterial inoculum was prepared according to standardized methods [26].

The bacterial strain was transferred from the maintenance medium into a tube containing the brain heart infusion (BHI) and kept at 35 °C for 18 h for strain activation. To isolate young colonies, we transferred aliquots into a Petri dish containing Muller-Hinton agar, and incubated it at 35 °C for 24 h. Then, 4–5 colonies were transferred into a tube containing 5 mL of sterile saline solution (0.85%) followed by homogenization in a vortex mixer for 15 s.

The turbidity of the cell suspension was adjusted to obtain an optical density similar to the 0.5 McFarland standard, which corresponds to a suspension containing approximately 1 to 2 × 10^8^ cells mL^−1^. Subsequently, this solution was diluted (1:10) to obtain an inoculum containing 10^7^ cells mL^−1^.

The antimicrobial activity was examined using the broth microdilution technique [26] with modifications. The method comprised preparation of successive dilutions of each treatment to be assessed—CCE, EAF, Ca(OH)_2_ solution, Ca(OH)_2_ solution with CCE, and 2% CHX—in a liquid culture medium, inoculation of the bacteria, and after incubation, interpretation of the result for determining the MIC.

The treatments were placed in 96 well microplates (TPP Cultilab; Campinas, SP, Brazil). The treatments were diluted to obtain a final concentration in each well from 166.50 to 2.6 μg mL^−1^ for the solutions, and from 1 to 0.01% for the CHX. Then, 100 μL (0.1 mL) of the inoculum suspension to the wells was added. The columns were filled with suspensions as follows: microdilutions of the treatments, culture media control (negative control), controls for the treatments, and control for *E. faecalis* viability (positive control). Five test wells were repeated in each group.

The plates were incubated at 35 °C for 24 h and were read visually to determine the MIC. When bacterial growth was confirmed, suspensions from each well were transferred into Petri dishes identified and containing nutrient agar. After incubation at 35 °C for 48 h, the presence or absence of bacterial growth was evaluated. The MIC was regarded as the lowest concentration in the series of dilutions, which did not permit the growth of the susceptible bacteria [9]. So, to determine the MIC, we compared the bacterial growth in each well with that in the positive control and that observed in the nutrient agar dishes.

The concentration of the powder products tested was determined by calculating the concentration of the stock standard solution, by using the following formula: amount of product (mg)/volume of the vehicle (mL); thus, 550 mg/1.5 mL ≈ 333 mg mL^−1^.

Calculation of constant concentrations and volumes was used to determine the initial concentration of the products tested (concentration of the stock standard solution). To calculate the concentration of each dilution, we used the equation of constant concentration and volume (C1 × V1 = C2 × V2). We used the same volume in all wells, and thus, the dilution in each subsequent well was half of that in the previous well; we successively filled all wells in this manner (with the lower concentration tested).

## Results

The MICs of the treatments are shown in Table 1.

**Table 1:** Minimum inhibitory concentrations (MICs) of treatments against *Enterococcus faecalis*

Treatments	MIC (mg mL^−1^)						
	166.50	83.25	41.62	20.81	10.40	5.20	2.60
CCE	+	+	+	+	+	+	+
EAF	−	−	−	+	+	+	+
Ca(OH)_2_	−	−	+	+	+	+	+
Ca(OH)_2_ + CCE	−	+	+	+	+	+	+

	MIC (%)						
	1.00	0.50	0.25	0.12	0.06	0.03	0.01
CHX	−	−	−	+	+	+	+

(+) bacterial growth, (−) no bacterial growth, *CCE* concentrated crude extract, *EAF* ethyl acetate fraction, *Ca(OH)*_*2*_ calcium hydroxide, *CHX* chlorhexidine digluconate

The CCE did not inhibit bacterial growth at any of the concentrations tested. Contrary, all the other acted at various concentrations. The EAF inhibited bacterial growth at three highest concentrations (166.50, 83.25, and 41.62 mg mL^−1^). The same was observed with 1.00, 0.50, and 0.25% of CHX solutions. Ca(OH)_2_ + CCE showed antimicrobial activity only at its highest concentration, 166.50 mg mL^−1^.

## Discussion

This study aimed to compare the antibacterial activity of the CCE and EAF from *P. major*, Ca(OH)_2_, Ca(OH)_2_ + CCE, and CHX against *E. faecalis.* We observed satisfactory antimicrobial activity with the treatments, in particular the EAF, but the same was not observed with CCE.

Extensive research has been performed to identify substances that enable elimination of microorganisms from the root canal. However, therapeutic failure continues to occur in patients with persistence of the infection. Thus, we selected *E. faecalis*, a highly resistant bacterium, for this study [3].

The antimicrobial activity of substances described in previous studies, such as Ca(OH)_2_ [4–6] and CHX [6], including their combinations against this bacterium warrants further clarifications [7]. Ca(OH)_2_ is one of the most frequently used drugs in microbiological studies and in the field of Endodontics [4]. Some authors suggest that the effect of this drug against *E. faecalis* is low [6, 27]. Treatment with Ca(OH)_2_ showed antibacterial activity only at two of its highest concentrations (166.50 and 83.25 mg mL^−1^). This may be explained by the fact that *E. faecalis* is not sensitive to alkaline environment [28]. A study showed that the release of hydroxyl ions does not have sufficient antimicrobial activity against microorganisms [29].

The 2% CHX has been considered superior to Ca(OH)_2_ as an intracanal medication [30]. A recent study showed that the antimicrobial activity of CHX is increased when used in combination with Ca(OH)_2_ [31]. Our results showed that CHX was effective against *E. faecalis* at concentrations of 1, 0.5, and 0.25%. Our findings are consistent with those that observed antimicrobial effect at 1 and 0.5% of CHX [32]. Arias-Moliz et al. [33] found low efficacy of 2% CHX in the elimination of *E. faecalis*, while other authors reported that 2% CHX used as an intracanal drug eliminated *E. faecalis* from the root canal system [34].

In the recent years, studies are being performed to identify and examine active principles from medicinal plants for use in dentistry against *E. faecalis*. *Morinda citrifolia* [8], *Triphala* [9], berberina [10], *Syzygium aromaticum*, *Ocimum sanctum* and *Cinnamomum zeylanicum* [11], *Myrtus communis* [12], *Ferula gummosa* [13], and oregano [14] have shown promising results. We used *P. major* in this study; the antimicrobial potential of some of the bioactive components present in the different parts of this plant has been examined in a previous study [15]. Among these components, tannins, in particular the condensed tannins, have diverse important biological effects such as antimicrobial, antiseptic, and antifungal effects [23]. Tannins are phenolic compounds soluble in water, with molecular weight about 500–3000 Da, with ability to form insoluble complexes in water with proteins, gelatins and alkaloids [35]. This compound is responsible for the sensation of astringency caused by certain food, due to the interaction with the salivary proline-rich proteins (PRPs) in the oral cavity. The tannin-induced interaction and/or precipitation of proteins occur, probably, by means of hydrogen bridges between the phenolic groups of tannins and attachment sites of proteins [36]. Metals and enzymes easily influence the oxidation of tannins, thus the searching for these plant compounds can be performed by adding, for example, ferric chloride, lead acetate and gelatin, which promote the color change of the solutions and the formation of precipitates [37].

Few studies have evaluated the antimicrobial potential of this plant and its application; therefore, investigations are required to determine the antibacterial activity of *P. major* extracts against *E. faecalis* and to detect the concentrations responsible for the inhibition of bacterial growth, including research in dentistry. Our results showed that the CCE had a low potential of inhibiting *E. faecalis*, such as the combination of Ca(OH)_2_ + CCE. However, promising results were observed with the EAF.

We used the microdilution technique, because this method was used in studies performed examining the effect of *M. citrifolia* as an irrigant solution [8], in an analysis of *Triphala* [9], and berberine [10] against *E. faecalis*. The MIC has been used in dentistry studies involving the analysis of antimicrobial effect of plant products [9, 10]. In this study, the MIC was determined by observing the growth of *E. faecalis* in Petri dishes containing nutrient agar.

Although the compounds have been extracted from the same plant, different effects were found on *E. faecalis*. We suggest that this may have occurred because of the CCE have all the secondary metabolites present, while the EAF was a fraction which aimed the action of phenolic compounds, especially the tannins. Our results showed that the EAF from *P. major* inhibited the growth of *E. faecalis*. The extract from this plant may be a promising alternative treatment for fighting microorganisms involved in endodontic infections.

We have registered a patent (BR 10 2012 028509 6) for using this plant for its wound healing, anti-inflammatory, and antimicrobial activities in dental care.

Limitations of this study are due to the fact that we have evaluated the action of different substances on only one microorganism. More studies are required to be verified behavior through the biofilm and tissue effects.

## Conclusions

The CCE did not show antimicrobial activity against *E. faecalis*, and the EAF was the most effective among the three highest concentrations studied.

## Abbreviations

CCE: concentrated crude extract (CCE)
EAF: ethyl acetate fraction (EAF) of CHX: 2% chlorhexidine digluconate
MIC: minimum inhibitory concentrations
BHI: brain heart infusion
